# Purification of Polybutylene Terephthalate by Oligomer Removal Using a Compressed CO_2_ Antisolvent

**DOI:** 10.3390/polym11071230

**Published:** 2019-07-23

**Authors:** Wen Yu, Jian-Hao Huang, Chung-Sung Tan

**Affiliations:** Department of Chemical Engineering, National Tsinghua University, Hsinchu 30013, Taiwan

**Keywords:** polybutylene terephthalate, cyclic dimer, cyclic trimer, 1,1,1,3,3,3-Hexafluoro-2-propanol, compressed CO_2_, antisolvent

## Abstract

In this study, the cyclic oligomers in the highly chemically resistant polyester polybutylene terephthalate (PBT) were effectively removed using a compressed CO_2_ antisolvent technique in which 1,1,1,3,3,3-hexafluoro-2-propanol (HFIP) was used as the solvent. In addition to the oligomers, tetrahydrofuran was completely removed because of its low molecular weight and liquid state. The effects of the operating variables, including temperature, pressure, and the PBT concentration in HFIP, on the degree of removal of the oligomers were systematically studied using experimental design and the response surface methodology. The most appropriate operating conditions for the purification of PBT were 8.3 MPa and 23.4 °C when using 4.5 wt % PBT in HFIP. Under these conditions, the cyclic trimers and dimers could be removed by up to 81.4% and 95.7%, respectively, in a very short operating time.

## 1. Introduction

Polybutylene terephthalate (PBT) is an important commercial engineering thermoplastic with widespread applications in electronics, automobile parts, and the mechanical industry because of its excellent mechanical and processing properties [1,2,3,4]. PBT can be produced by the polycondensation of dimethyl terephthalate or terephthalic acid with 1,4-butanediol. Commercial PBT generally contains some cyclic oligomers (e.g., cyclic dimer and trimer) resulting from the incomplete reaction and the byproduct tetrahydrofuran (THF). The presence of the oligomers and THF causes undesirable effects in the subsequent processing of PBT, such as dyeing and injection molding. Furthermore, it is challenging to remove the oligomers and THF, which are wrapped in the PBT chains. 

A number of research groups have investigated the removal of oligomers from polyesters [5,6,7,8]. The extraction of oligomers from a similar thermoplastic, poly(ethylene terephthalate) (PET), using a liquid–solid Soxhlet extractor resulted in the removal of oligomers but required the use of a possibly carcinogenic solvent, 1,4-dioxan, and extremely long extraction times because of the nonporous nature of the materials [7,8]. Inagaki et al. [6] used subcritical water to extract soluble oligomers from PET at 5 MPa and 200 °C in 10 min, which reduced the oligomer content by 16%. Weijers et al. [5] proposed the use of supercritical CO_2_ to extract cyclic oligomers from PBT. The extraction efficiency for cyclic dimers was 34.8%, achieved by treatment at 25 MPa and 230 °C for 2.5 h under a 31.5 mL/min flow of CO_2_. Even though supercritical CO_2_ has no surface tension, a high extraction efficiency could not be achieved effectively because the absorption of CO_2_ by PBT is not sufficiently high, and, consequently, the expansion of PBT is insufficient to allow CO_2_ to enter the nonporous PBT. However, the addition of pentane (1 mol %) increased the solubility of the cyclic dimer in the modified supercritical CO_2_ solution, resulting in an increase in the extraction rate to 74.6%. However, these methods require severe operating conditions, such as high temperatures and pressures, as well as long operating times, which result in high energy consumption and costs and, consequently, limit their industrial applications. In addition, the extraction efficiency of the oligomers requires further improvement. Hence, it is necessary to develop a greener method to purify PBT.

Compressed fluids are effective antisolvents for the generation of particles with a narrow particle size distribution from solutions containing the solid solute [9,10,11,12,13,14,15,16]. To apply antisolvent techniques, the following requirements are in general to be met: (1) the solute must be insoluble or significantly less soluble in the compressed fluid, and (2) the organic solvent must expand large enough after the dissolution of the compressed fluid to significantly reduce the interaction force between solute and solvent. The expansion weakens the interactions between the solvent and solute, resulting in immediate precipitation of the solute (usually in few seconds). The compressed fluid can be in a liquid, gaseous or supercritical state, depending on the operating temperature and pressure.

The interactions between the solvent and solutes caused by solvent expansion can be tuned by varying the operating temperature and pressure. The degree of expansion is generally increased at low temperatures and high pressures [17,18]. Obviously, solutes with higher molecular weights are precipitated before those with lower molecular weights when a mixture of solutes is present in a solvent; as a result, the fractionation of solutes can be achieved [19,20,21,22]. CO_2_ has a moderate critical temperature and pressure (31.1 °C and 7.4 MPa) [23] and can be dissolved in most organic solvents for solvent expansion. Moreover, CO_2_ is readily available, inexpensive, nontoxic, and easy to separate from the precipitated materials by depressurization [24,25]. Consequently, it is frequently used as an antisolvent.

To apply the compressed CO_2_ antisolvent technique for the purification of PBT by removing oligomers and THF, an appropriate solvent should be chosen first. It is well known that PBT is a highly chemically resistant polyester and cannot be dissolved in most organic solvents. Fortunately, 1,1,1,3,3,3-hexafluoro-2-propanol (HFIP) has been found to dissolve PBT at room temperature, although not significantly [26], and it is also miscible with compressed CO_2_ because of the high affinity of the fluorine atom for CO_2_ [27,28]. Though HFIP is a fluorinated solvent, it is better to be used than 1,4-dioxan with the concern of carcinogenicity. Nevertheless, HFIP should be collected after the operation for the cycling use purpose to avoid causing environmental impact. HFIP can be expanded by 1.5 to 3.5 times its original volume after mixing with compressed CO_2_ at 40 °C from 4.5 to 7.2 MPa. Therefore, HFIP was chosen as the solvent and compressed CO_2_ as the antisolvent for the purification of PBT. Because the molecular weight of PBT is higher than those of the cyclic oligomers including cyclic dimers and trimers and the byproduct THF (THF with very low melting point as −108.4 °C cannot be precipitated in general because of its liquid state under the operating conditions), the determination of the oligomer concentration in PBT can be used to determine the degree of oligomer removal from the precipitated PBT. On the basis of the degree of removal, the feasibility of the proposed technique can be determined. It is known that the morphology and size of the precipitated solutes depends heavily on the operating conditions, including the solute concentration in the solvent, the temperature, and the pressure [10,29,30,31]; thus, another objective of this study is to examine the effects of the operating variables on the purification efficiency, as well as the morphology and size of the precipitated PBT, in a systematic fashion. 

## 2. Materials and Methods

PBT (*M*_w_ = 1200) was obtained from Chang Chun Petrochemical Co. (Taipei, Taiwan, the largest supplier of PBT in the world). Two methods to purify the PBT were tested in this study, which were the extraction of cyclic oligomers from PBT by HFIP (≥99.8%, Sigma–Aldrich, St. Louis, MO, USA) containing compressed CO_2_ technique and the compressed CO_2_ antisolvent technique. For the extraction of cyclic oligomers from PBT by HFIP containing compressed CO_2_ technique, PBT was placed in a high-pressure contactor and allowed to contact with three times its weight of HFIP containing compressed CO_2_ at the desired temperature and pressure. The reason for using HFIP containing compressed CO_2_ was to ensure that more of the oligomers could be dissolved in HFIP than PBT. If PBT came in contact with HFIP at normal conditions, both PBT and oligomers were dissolved into HFIP, causing no change of oligomers’ contents in the remained solid PBT. After a certain contact period, the solid PBT was then collected by removing the liquid HFIP solution containing the dissolved PBT, oligomers, and THF. The collected particles were dried and analyzed using NMR spectroscopy (Bruker Avance 500, Billerica, MA, USA). For NMR sample preparation, 30 mg of sample was dissolved in 3 mL CDCl_3_:CF_3_COOD (8:1).

**Figure 1:**
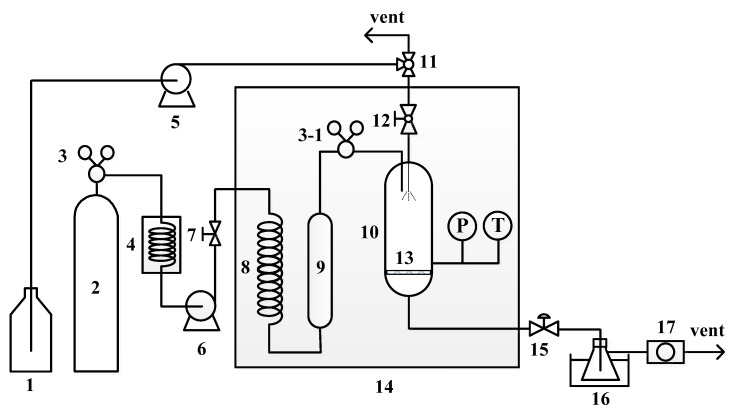
Schematic diagram of apparatus for compressed CO_2_ antisolvent operation: (1) PBT/HFIP solution tank, (2) CO_2_ cylinder, (3) regulator, (4) cooler, (5) HPLC pump, (6) high-pressure pump, (7) needle valve, (8) coil, (9) surge tank, (10) precipitator, (11) three-way valve, (12) ball valve, (13) filter, (14) oven, (15) metering valve, (16) cold bath, and (17) wet gas meter.

Figure 1 shows a schematic of the apparatus used for the purification of PBT using the CO_2_ antisolvent technique. In a typical trial, CO_2_ (≥99.5%, Boclh Industrial Gases Co., Taipei, Taiwan) at the desired temperature and pressure and a constant flow rate of 2 L/min was continuously introduced into the precipitator by a high-pressure piston pump (Milton Roy, Ivyland, PA, USA, MD46). The precipitator was placed in an oven (Firstek, New Taipei City, Taiwan, RI-600), so the operation temperature could be controlled and maintained. After a steady state had been reached, the PBT in the HFIP solution, prepared by dissolving a certain amount of PBT into HFIP at normal conditions with the assistance of ultrasound, was injected into the precipitator at a flow rate of 10 mL/min. The solution droplets and films thus made contact with the compressed CO_2_. PBT was precipitated first because its solubility in the CO_2_-containing HFIP was lower than those of the oligomers and THF. THF should not be precipitated because of its complete miscibility with HFIP. After about 30 s, the injection of the mixed solution was stopped and the compressed CO_2_ was allowed to flow through the precipitator for another 2 h to remove the HFIP retained on the precipitated PBT. The system was then depressurized to atmospheric pressure, and the precipitated particles were collected for further analysis of the PBT composition.

In the purification study using the compressed CO_2_ antisolvent technique, an NMR spectrometer was also used to determine the cyclic dimer concentration in the collected PBT, just as for the extraction of oligomers from PBT by HFIP containing compressed CO_2_ technique. Because not only the cyclic dimer but also the cyclic trimer were present in the original PBT, to obtain more precise information about the oligomer contents of the precipitated PBT, a proprietary high-performance liquid chromatography (HPLC) measurement technique, including the column and operating conditions, developed by Chang Chun Petrochemical Co. was employed as well in this study. The analysis of the oligomers in PBT was carried out as follows: PBT was dissolved in a mixture of HFIP and chloroform (99.8%, Sigma–Aldrich) at 50 °C, which was then cooled to ambient temperature. Subsequently, propionitrile was added to precipitate the polyester. After the polyester had been completely precipitated, the supernatant was removed and analyzed using HPLC (LDC Analytical, Waltham, MA, USA, Constametric 3200).

Scanning electron microscopy (SEM) images were obtained using a JSM-5600 (JEOL, Tokyo, Japan). The particles size distribution was determined by using the OPTIMAS 5 imaging analysis software (Houston, TX, USA). The sizes of at least 200 particles were recorded in order to obtain the average particle size and standard deviation.

## 3. Results and Discussion

**Figure 2:**
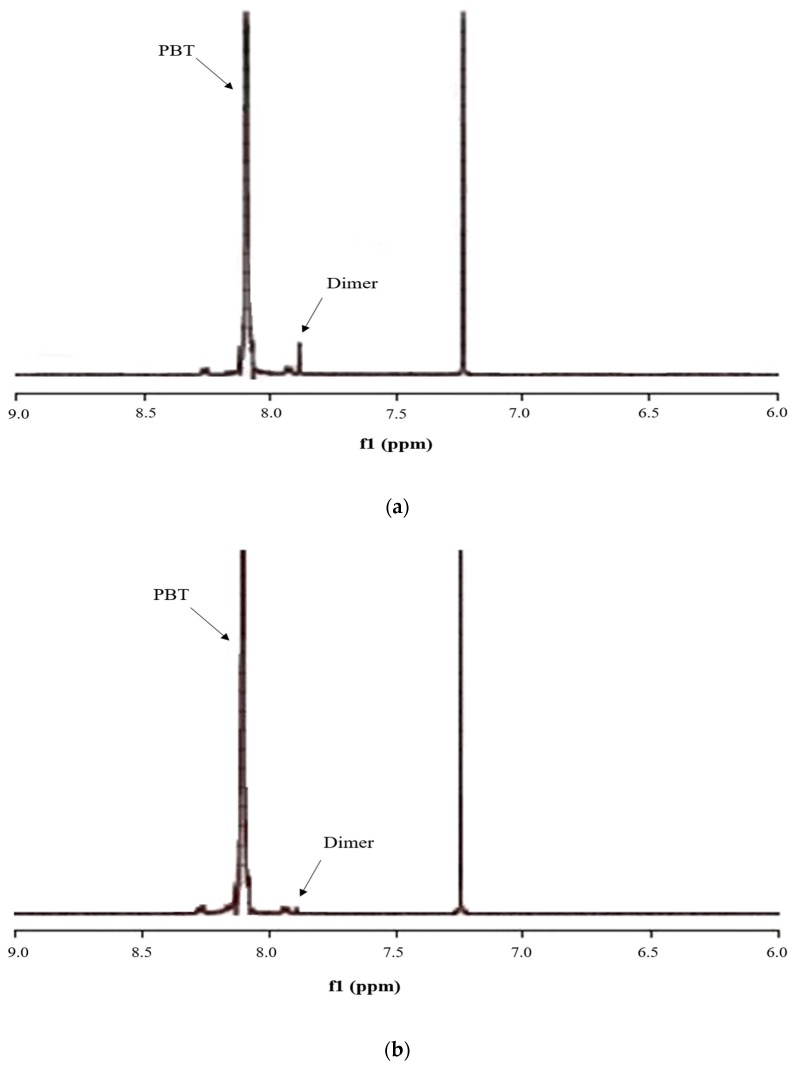
^1^H-NMR spectra of (**a**) initial PBT and (**b**) that collected after extraction by HFIP containing compressed CO_2_.

For the extraction of cyclic dimers from solid PBT by HFIP containing compressed CO_2_, the proton NMR peaks for PBT and the cyclic dimer [4] before and after the extraction with HFIP solution for a typical operation are shown in Figure 2. It can be seen that the intensity of the peak corresponding to cyclic dimer was decreased, indicating that the cyclic dimer in PBT was dissolved in HFIP to a greater extent than PBT because of its lower molecular weight. Consequently, the amount of cyclic dimer in PBT was reduced after PBT was contacted with HFIP containing compressed CO_2_.

Table 1 shows the removal efficiency of cyclic dimer at various temperatures, pressures and contact periods. The percentage removal was determined by calculating the area of the peak corresponding to the cyclic dimer in the NMR spectrum. It is clearly seen that the purification efficiency by HFIP without containing compressed CO_2_ could not be comparable with those by HFIP containing compressed CO_2_, indicating that the presence of compressed CO_2_ was essential to remove more cyclic dimer from PBT. To verify the role of the presence of compressed CO_2_ in HFIP, helium instead of CO_2_ was used in the experiment, the cyclic dimer removal efficiency was found to be the same as the use of HFIP itself, shown in Table 1. The benefits of the presence of compressed CO_2_ in HFIP might have been due to the solubility of cyclic dimer being higher than that of PBT in HFIP containing compressed CO_2_, the diffusion coefficient of a CO_2_-expanded solvent was increased [32,33] and dilation of PBT was caused by absorption of CO_2_. It is also seen from Table 1 that that longer contact periods and higher pressures favored the removal of the cyclic dimers. For example, at 13.9 MPa, 38% of the cyclic dimers was removed after 8 h. This is significantly higher than the 24.8% extraction of cyclic dimers obtained after treatment for 8 h at 6.9 MPa. In contrast, the temperature was found to have little effect between 40 and 50 °C. Because that it took such a long contact period but only to result in such low cyclic dimer removal percentages, the extraction method was not suggested to use for the PBT purification purpose. An attempt to obtain PBT possessing lower concentrations of cyclic oligomers using the compressed CO_2_ antisolvent technique was therefore applied.

In the compressed CO_2_ antisolvent process, droplets or films of the HFIP solution containing PBT, cyclic oligomers and THF came into contact with compressed CO_2_. The PBT, which has a higher molecular weight than the oligomers, should be precipitated first because of its lower solubility in the expanded HFIP solution after the dissolution of CO_2_ in HFIP. With this concern, the temperature should be reduced and the pressure increased to increase the degree of expansion of HFIP and reduce the interactions between the oligomers and the solvent. Furthermore, on precipitation of the cyclic oligomers, the cyclic trimers should be precipitated to a greater degree than the cyclic dimers because of the higher molecular weight of the former. Therefore, the removal percentage of the cyclic trimers can be used as a metric for the total oligomer removal.

Concerning the energy required for HFIP expansion, gaseous CO_2_ at 6.4 MPa and 25 °C was first used as the antisolvent, just as in our previous study for precipitation of spherical poly(methyl methacrylate) from toluene [34]. Unfortunately, only a small amount of solid PBT was found to precipitate, and this showed signs of severe coalescence, indicating that no significant nucleation occurred with gaseous CO_2_.

Because the compressed gaseous CO_2_ did not act as an appropriate antisolvent, an increase in the pressure or reduction in the temperature were required to induce greater solvent expansion and reduce the interactions between PBT and HFIP. To allow more PBT than oligomers to precipitate, an experimental design and response surface methodology with 2^3^ factors, 6-axis point experiment, and 6-center point experiment were used to study the effects of the operating pressure and temperature and the PBT concentration in HFIP on the removal efficiency of cyclic trimers from the precipitated PBT systematically. The selected high and low levels for the pressure (*X*_1_), temperature (*X*_2_), and PBT concentration in HFIP (*X*_3_) were 7.6 to 8.3 MPa, 25 to 30 °C, and 4% to 5%, respectively.

**Figure 3:**
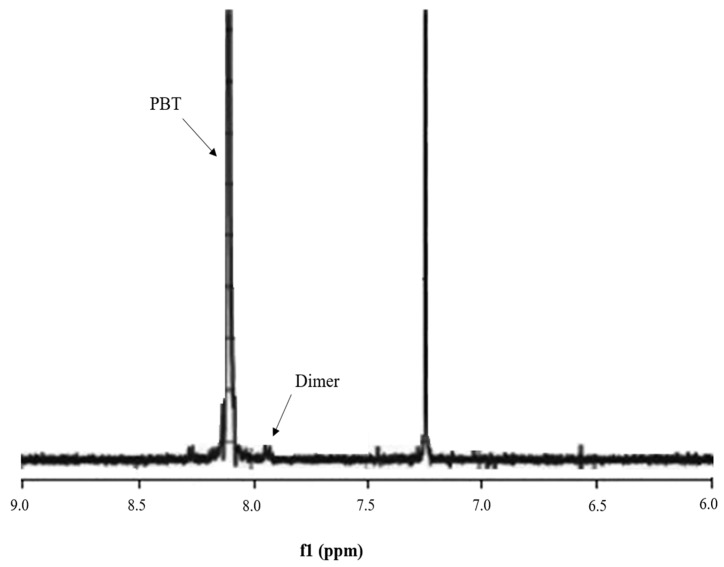
^1^H-NMR spectra for PBT collected after the compressed CO_2_ antisolvent treatment.

**Figure 4:**
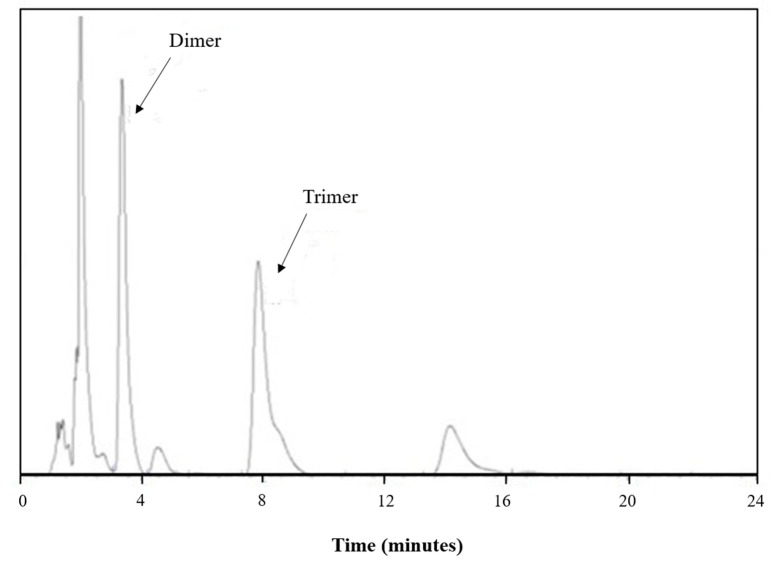
HPLC traces of initial PBT.

For a typical run using the CO_2_ compressed antisolvent technique with a pressure higher than 7.6 MPa, the peak corresponding to the cyclic dimer was found to be quite small, as shown in Figure 3, indicating that the cyclic dimer could be removed effectively. A higher removal efficiency of the cyclic dimer is believed to be resulted from a higher expansion of the solvent at a higher pressure. However, not only the cyclic dimer but also the cyclic trimer were present in the original PBT. The analysis of presence of the dimer and trimer in the collected PBT using HPLC was also performed in this study. The HPLC traces of the initial PBT are shown in Figure 4, indicating that the initial PBT contained 0.48 wt % cyclic dimer and 1.58 wt % cyclic trimer. The cyclic oligomers removal efficiency of the antisolvent treatment of PBT was measured twice under the same operating conditions to ensure repeatability. The difference between purification runs was less than 3.5%, indicating that the measured oligomer removal efficiency was representative and the process was reproducible and reliable.

**Figure 5:**
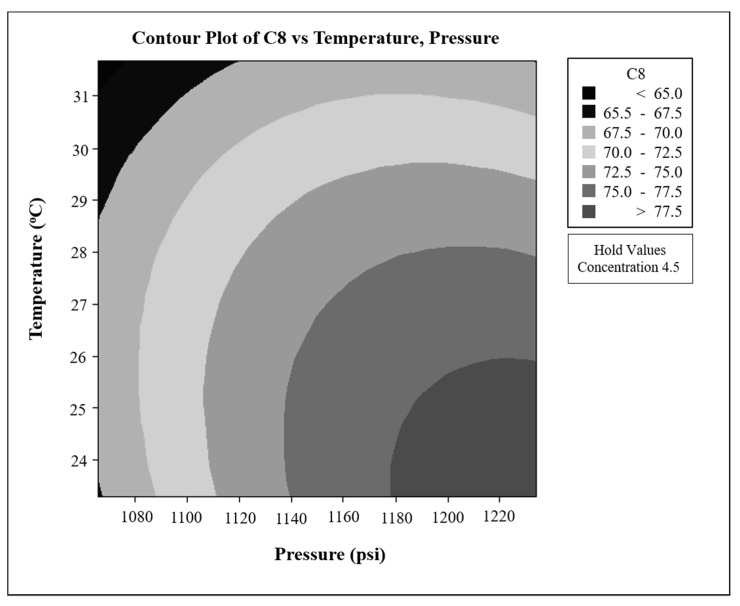
Cyclic trimer concentration dependence at various temperatures and pressures for a PBT concentration in HFIP of 4.5 wt %.

The removal efficiency of cyclic trimers from PBT and the analysis of variance (ANOVA) based on the experiment results are shown in Table 2 and Table 3, respectively. Figure 5 shows the effect of CO_2_ pressure and temperature on the cyclic trimer removal efficiency at a PBT concentration in HFIP of 4.5 wt %. It is clearly seen from Figure 5 that the greater solvent expansion resulting from the higher pressure and lower temperature is favorable to obtain PBT with a low content of the cyclic trimer because of more dissolving power towards oligomers by the CO_2_-expanded HFIP. The greater solvent expansion resulting from the higher pressure and lower temperature is favorable to obtain PBT with a low content of the cyclic trimer.

In the variance analysis, the sum of squares is the variation between the studied factors. The mean square of each factor is the sum of the squared sum divided by the degree of freedom. The F-value is the mean square of each factor divided by the mean square of the residual. At 95% confidence level, a *p*-value of less than 0.05 indicates significant effects of those variances. The ANOVA results in Table 3 indicate that pressure and temperature were both significant factors. This is because the pressure and temperature were the major factors influencing solvent expansion, resulting in the precipitation of the higher molecular weight molecule (PBT) first, whereas the oligomers and THF remained in solution.

A regression model equation with a coefficient of determination (R^2^) value of 0.952 was obtained from the polynomial regression model using Minitab 16, as shown below.

*Y* = 74.44 + 2.24*X*_1_ − 2.21*X*_2_ + 0.12*X*_3_ − 0.92*X*_1_^2^ − 0.83*X*_2_^2^ + 0.07*X*_3_^2^ − 0.08*X*_1_*X*_2_ − 0.04*X*_1_*X*_3_ − 0.04*X*_2_*X*_3_ (1)

**Figure 6:**
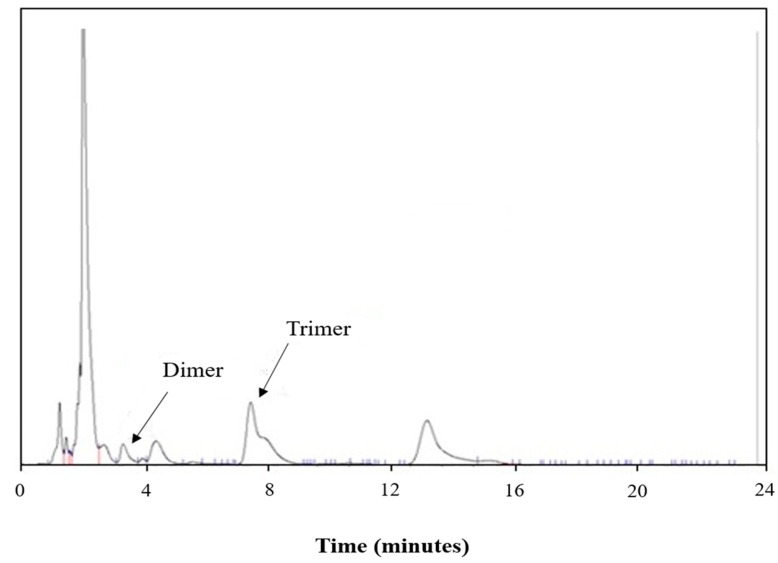
HPLC trace of PBT treated by the compressed CO_2_ antisolvent technique at 8.3 MPa and 23.4 °C and with a PBT in HFIP concentration of 4.5 wt %.

The optimum operating conditions using this regression model were found to be a pressure of 8.3 MPa, a temperature of 23.4 °C, and a PBT concentration in HFIP solution of 4.5 wt %. The predicted cyclic trimer removal rate was 80.2%, sufficiently close to the measured cyclic trimer removal rate of 81.4%, indicating the reliability of the regression model. In addition, the measured cyclic dimer removal rate under the optimum conditions was found to be 95.7%. The HPLC trace of the collected precipitated PBT is shown in Figure 6. As expected, a higher cyclic dimer removal rate was achieved when the cyclic trimer was also removed from PBT effectively. Moreover, no THF was detected in the precipitated sample. THF should be retained in the solution because of its low molecular weight and removed by the post-treatment of the precipitated solid with the flowing compressed CO_2_. Under the optimum operating conditions, the amount of the collected precipitated PBT, i.e., the yield of PBT, was found to be around 82% of the initially dissolved PBT in HFIP. It should be noted here that some of the precipitated PBT was retained on the wall of the precipitator, and there was a deviation in the yield of PBT in all experiments of ±6%; nevertheless, all the yields were greater than 70%. These results demonstrate that the proposed compressed CO_2_ antisolvent technique is effective in purifying PBT by removing oligomers and THF from PBT while giving PBT in a high yield. It is also noted here that the retained HFIP on the precipitated PBT removed by the compressed CO_2_ could be separated from gaseous CO_2_ easily after the reduction of pressure to atmospheric pressure. This recovered HFIP can then be mixed with the liquid solution for further use in this proposed purification process to achieve the recycling use purpose.

**Figure 7:**
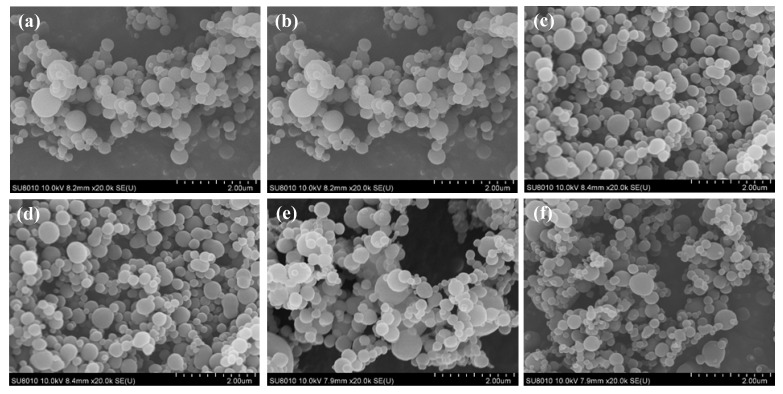
SEM images of the purified PBT particles at different conditions: (**a**) 25 °C, 7.6 MPa; (**b**) 25 °C, 7.6 MPa; (**c**) 25 °C, 8.3 MPa; (**d**) 25 °C, 9 MPa; (**e**) 27.5 °C, 8.3 MPa; and (**f**) 30 °C, 8.3 MPa.

The SEM images in Figure 7 show that the precipitated PBT had a spherical morphology when produced under various operating conditions. In addition, nano-sized PBT particles with a narrow particle size distribution were obtained. The average particle sizes of the PBT particles collected under the operating conditions shown in Figure 7 were similar, ranging from 239 to 259 nm.

## 4. Conclusions

Although PBT is a highly chemically resistant polymer, HFIP was identified as an effective solvent to dissolve PBT at normal conditions. In this study, the compressed CO_2_ antisolvent technique using HFIP as the solvent was verified to be more effective than the extraction of oligomers from PBT by the HFIP containing compressed CO_2_ technique for purifying PBT by removing the oligomers and THF. The compressed CO_2_ antisolvent process involved the injection of a solution consisting of PBT and HFIP into a compressed CO_2_ environment, causing the expansion of HFIP and resulting in the precipitation of PBT, leaving the oligomers and THF dissolved in HFIP. Post-treatment with compressed CO_2_ effectively removed the retained HFIP and any THF retained on the precipitated PBT. The operating pressure and temperature were observed to be the dominant operating variables. The optimum conditions for the removal of the cyclic oligomer were found to be 8.3 MPa, 23.4 °C, and 4.5 wt % of PBT in HFIP. Because of the low-temperature operation, the proposed technique uses significantly less energy to purify PBT than current processes. The removal rate of the cyclic trimer and cyclic dimer were 81.4% and 95.7%, respectively, with a yield of PBT of 82%. This removal rate of the cyclic dimer was significantly higher than 38% achieved by the extraction using HFIP containing compressed CO_2_ at 13.9 MPa after 8 h. Obviously, the purification by the compressed CO_2_ antisolvent process could be achieved within a very short operating time. Compared to existing techniques for the purification of PBT, the proposed method shows many advantages including high purification efficiency, low operating temperature, relatively low operating pressure, and short operating time. Therefore, not only the cost of purification but also the energy consumption can be reduced in the generation of highly pure PBT.

## Figures and Tables

**Table 1:** Removal efficiency of the cyclic dimer from PBT by the extraction method at various operating conditions.

Solvent	P (MPa)	T (°C)	Contact Time (h)	Removal Efficiency (wt %)
HFIP + CO_2_	6.9	40	2	15.3
HFIP + CO_2_	6.9	40	4	24.8
HFIP + CO_2_	6.9	40	8	28.2
HFIP + CO_2_	10.3	40	2	18.9
HFIP + CO_2_	10.3	40	4	28.8
HFIP + CO_2_	10.3	40	8	33.2
HFIP + CO_2_	13.1	40	8	38.1
HFIP + CO_2_	10.3	50	8	31.7
HFIP + CO_2_	13.1	50	8	36.1
HFIP	6.9	40	4	8.3
HFIP + He	6.9	40	4	8.2
HFIP	10.3	40	8	109
HFIP + He	10.3	40	8	11.2

**Table 2:** Removal efficiency of the cyclic trimer from PBT by compressed CO_2_ antisolvent technique.

Entry	P (MPa)	T (°C)	(PBT in HFIP) (wt %)	Removal Efficiency (wt %)
1	7.6	25.0	4.0	72.1
2	8.3	25.0	4.0	78.6
3	7.6	30.0	4.0	68.6
4	8.3	30.0	4.0	72.0
5	7.6	25.0	5.0	72.6
6	8.3	25.0	5.0	79.0
7	7.6	30.0	5.0	68.8
8	8.3	30.0	5.0	72.1
9	7.3	27.5	4.5	68.4
10	8.5	27.5	4.5	75.0
11	7.9	23.4	4.5	74.6
12	7.9	31.7	4.5	69.2
13	7.9	27.5	3.7	74.4
14	7.9	27.5	5.3	74.5
15	7.9	27.5	4.5	74.8
16	7.9	27.5	4.5	74.2
17	7.9	27.5	4.5	74.7
18	7.9	27.5	4.5	74.3
19	7.9	27.5	4.5	74.5
20	7.9	27.5	4.5	74.7

**Table 3:** Analysis of variance of the experimental design.

Source	Degrees of Freedom	Sum of Square	Mean Square	F-Value	*p*-Value
Regression	9	160.79	17.87	36.90	0.000
Linear	3	135.32	45.11	93.17	0.000
Pressure (*X*_1_)	1	68.56	68.56	141.62	0.000
Temperature (*X*_2_)	1	66.56	66.56	137.48	0.000
Concentration (*X*_3_)	1	0.20	0.20	0.40	0.539
Interaction	3	4.67	1.56	3.22	0.071
Pressure × temperature	1	4.65	4.65	9.61	0.011
Pressure × CO_2_ flow rate	1	0.01	0.01	0.02	0.882
Temperature × CO_2_ flow rate	1	0.01	0.01	0.02	0.882
Residual error	10	4.84	0.48		
Lack-of-fit	5	4.55	0.91	1.57	0.125
Pure error	5	0.29	0.06		
